# Supplementary material for: Individual variation in the functional lateralization of human ventral temporal cortex: Local competition and long-range coupling
Source: Imaging Neurosci (Camb). 2025 Mar 3;3:imag_a_00488. doi: 10.1162/imag_a_00488 (PMC11894816; doi:10.1162/imag_a_00488)
Supplement: Supplementary Material [file imag_a_00488-supp.pdf]

Supplementary Information:
Individual variation in the functional lateralization of human ventral temporal cortex: Local competition and long-range coupling

Nicholas M. Blauch[1,5,*], David C. Plaut[1,2], Raina Vin[1,4], and Marlene Behrmann[1,2,3]

[1] *Neuroscience Institute, Carnegie Mellon University*
[2] *Department of Psychology, Carnegie Mellon University*
[3] *Department of Ophthalmology, University of Pittsburgh*
[4] *Neurosciences Graduate Program, Yale University*
[5] *Department of Psychology, Harvard University*
[*] *Corresponding author: nblauch@fas.harvard.edu*

1 Group category-selective maps

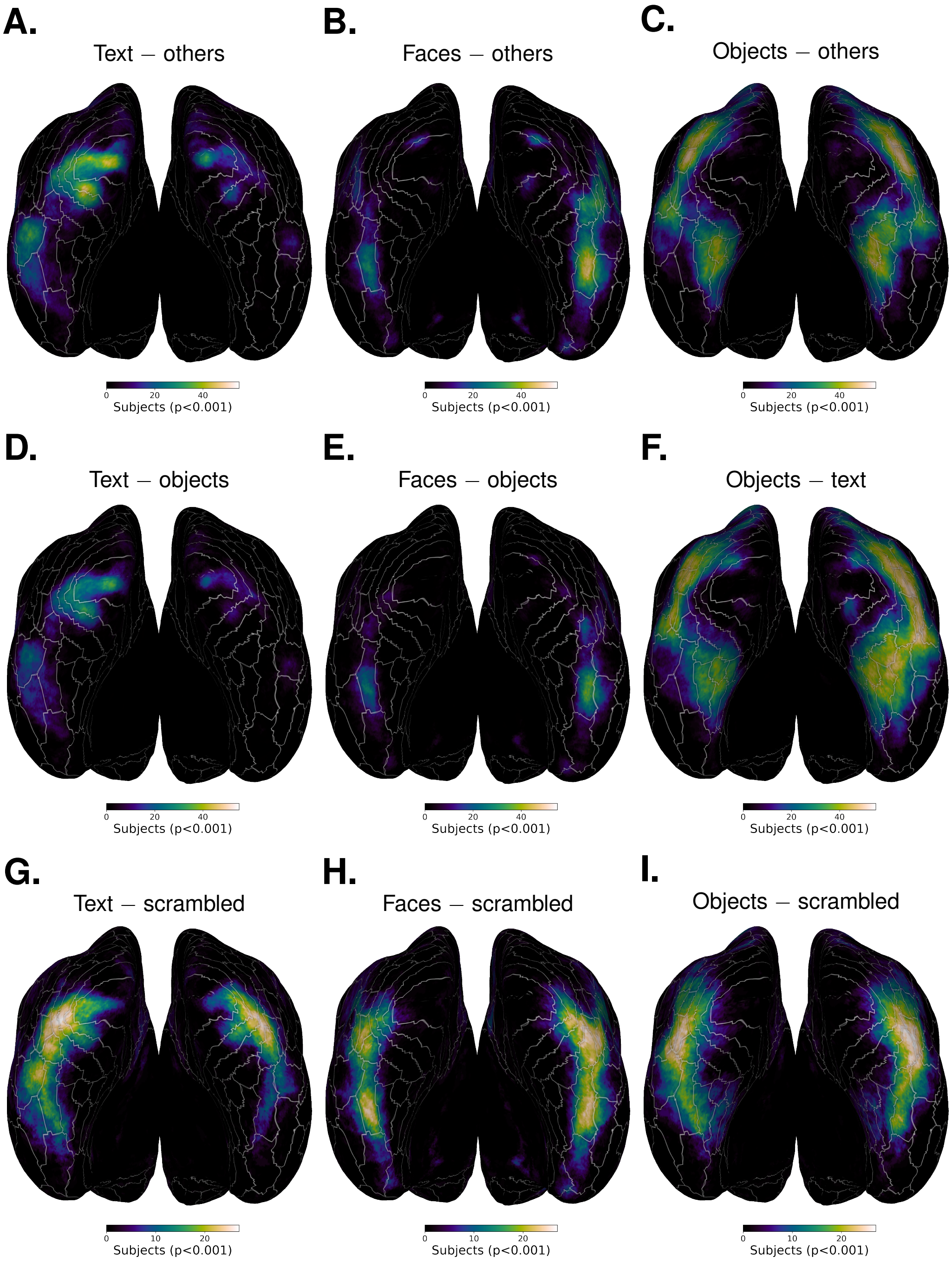

Figure S1: Group selectivity maps for relevant contrasts in the main experiment. Each map shows the number of participants that yielded significant selectivity for a given vertex in group surface space, where significance was determined as the selectivity value having $p<0.001$. Note: 55 participants were scanned, but only 28 saw the scrambled condition used in the second row.

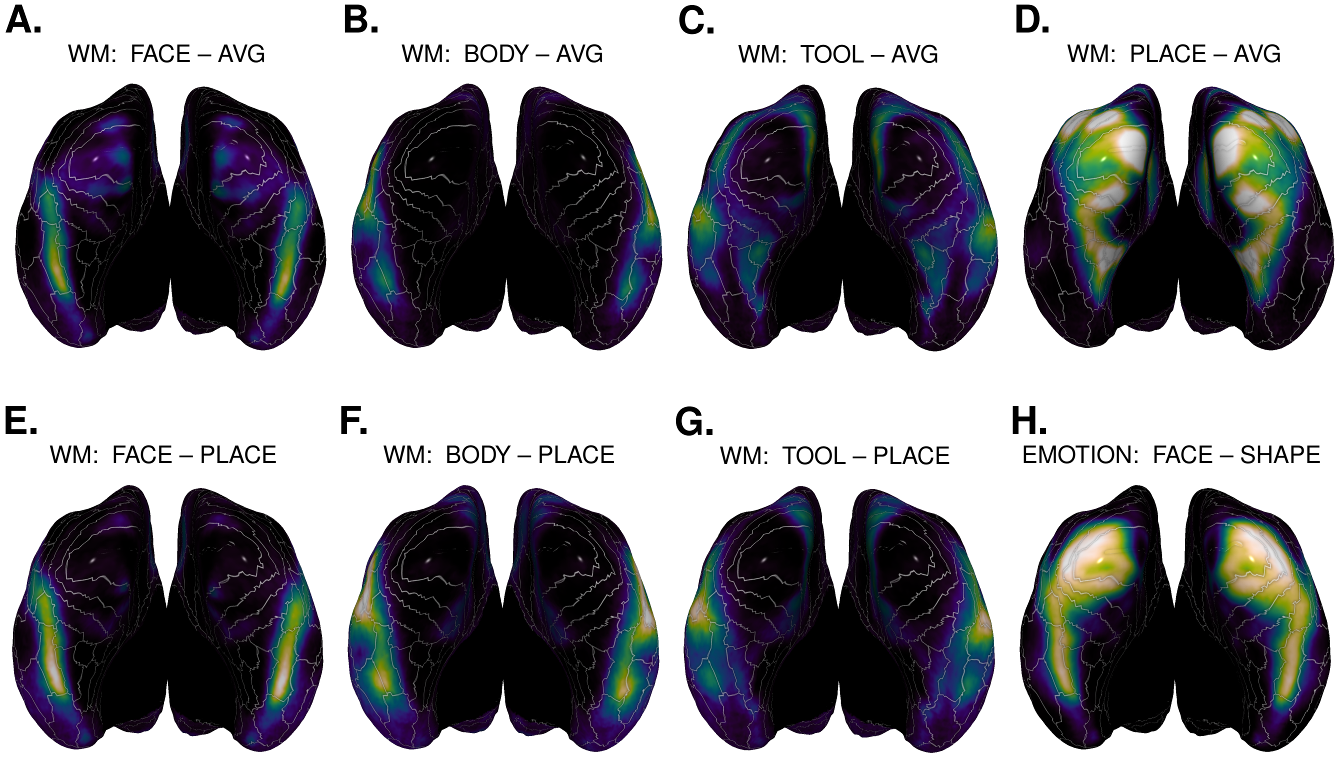

Figure S2: Group selectivity histograms for HCP contrasts. Color map goes from 0 to 700 participants showing selectivity ($p < 0.001$).

2 Overlap of category-selective responses

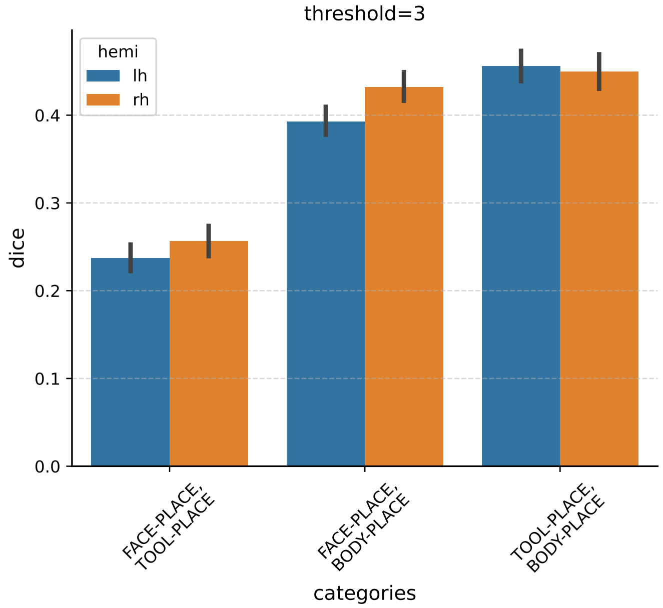

Figure S3: Overlap of category-selective regions in VTC, using the HCP WM contrasts of faces, tools, and bodies against places. A threshold of 3 was used to binarize the contrast maps before computing the dice coefficient.

3 Laterality using different metrics of selectivity

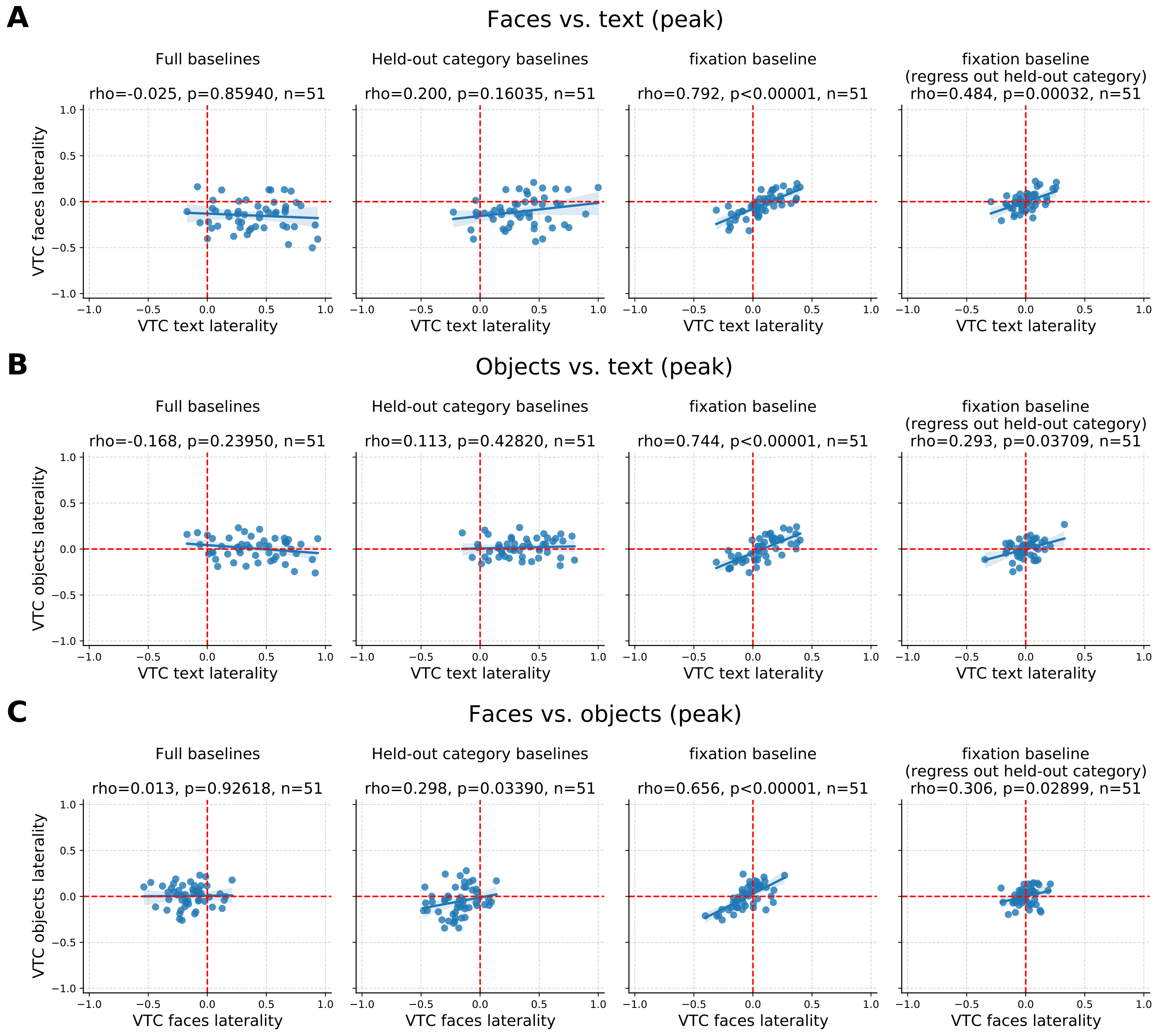

Figure S4: LI correlations using peak selectivity

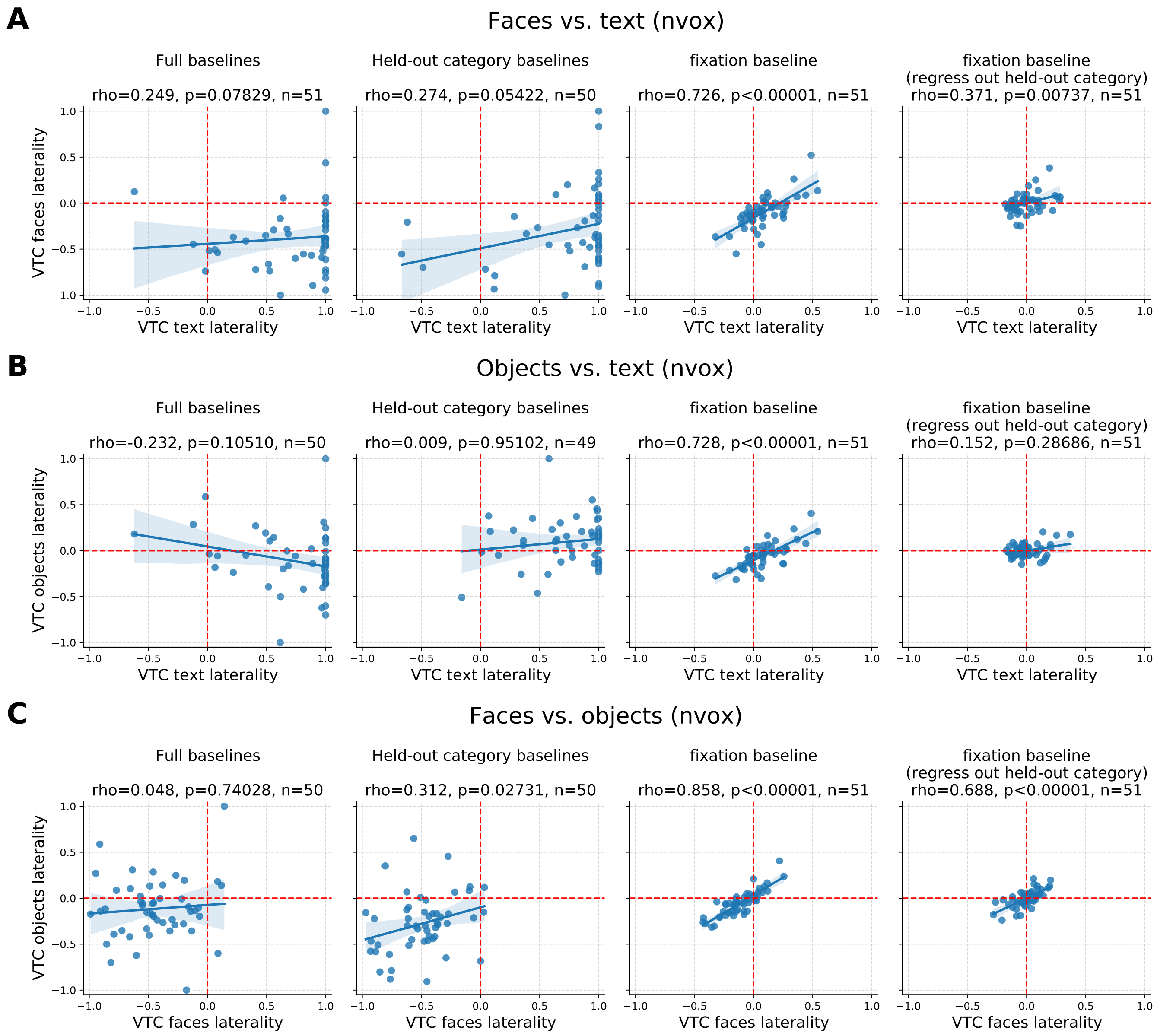

Figure S5: LI correlations using the number of selective voxels as a selectivity metric

4 Individual category-selective maps

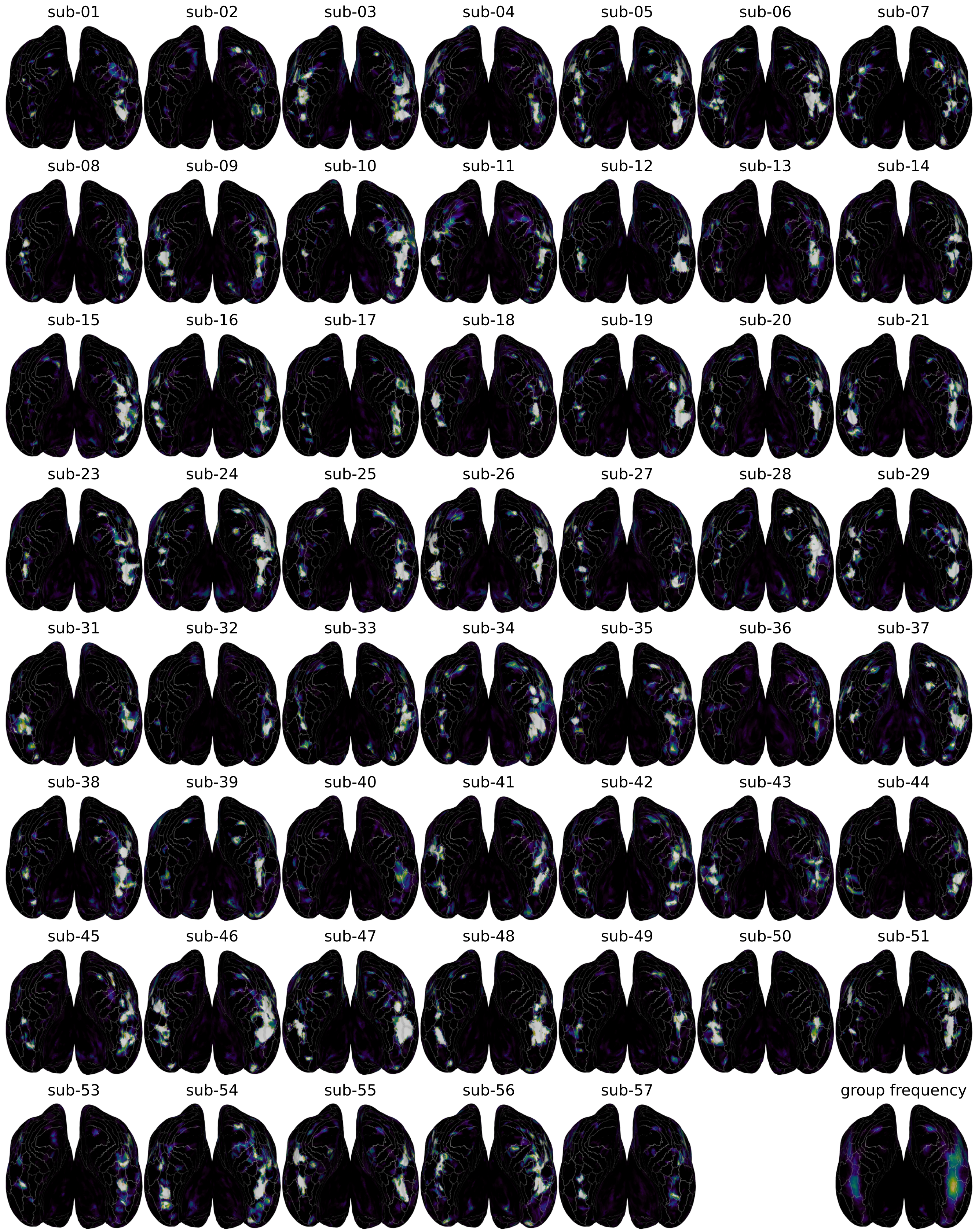

Figure S6: Individual face-selective maps for all subjects in our in-house experiment. The same approach as used in the main text (Figure 4D) was used to generate these maps, except all 5 runs (rather than even or odd) were used.

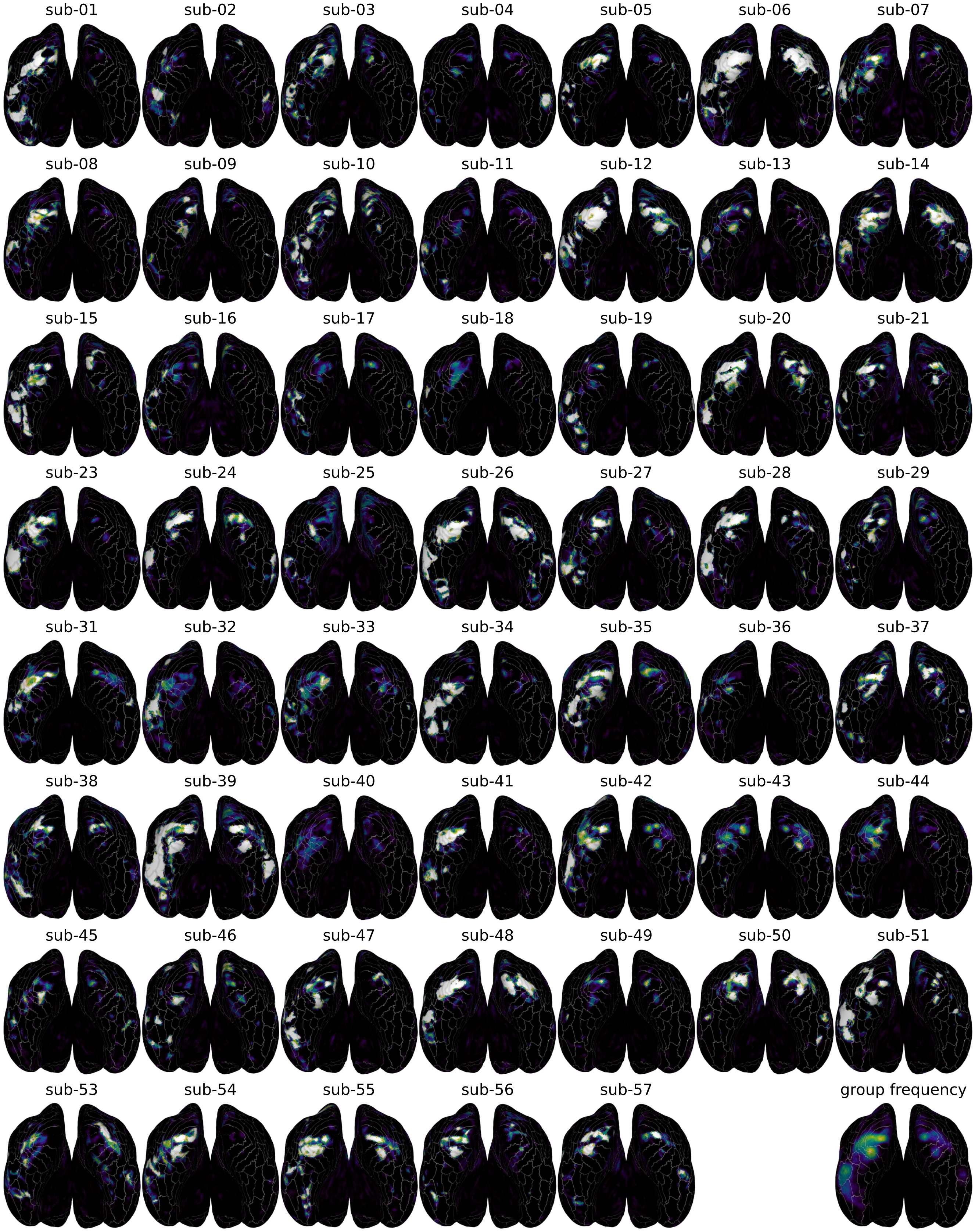

Figure S7: Individual text-selective maps for all subjects in our in-house experiment. The same approach as used in the main text (Figure 4D), except all 5 runs (rather than even or odd) were used.

5 Additional contrasts for parcel-level LI correlations in HCP

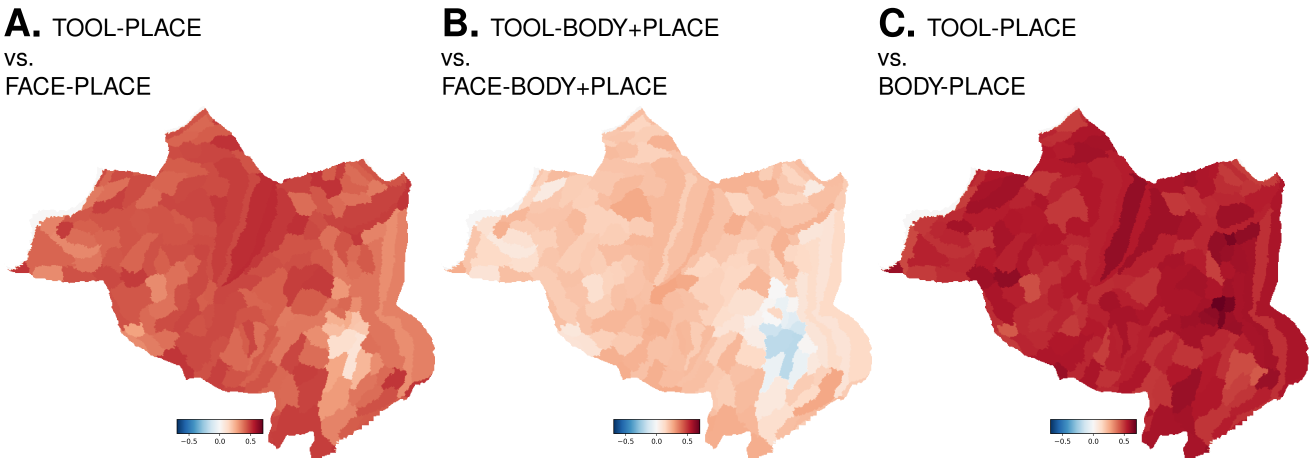

Figure S8: Within-experiment parcel-level LI correlations in HCP. This scenario more closely mimics the approach used in the main experiment.

6 Long-range face coupling analyses

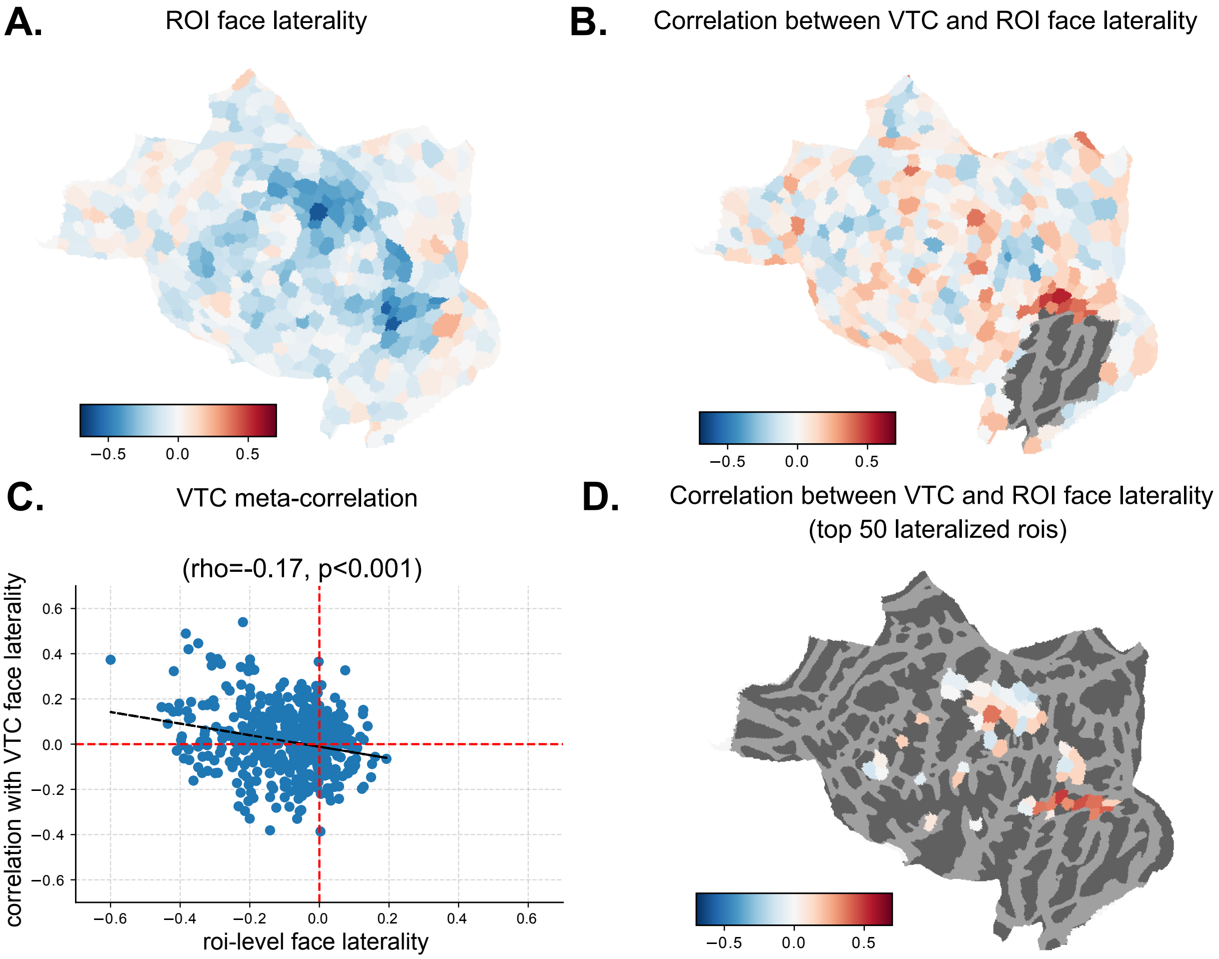

Figure S9: Data-driven assessment of whole-brain LI coupling of face selectivity in the main experiment. **A.** Mean of individual atlas parcels across participants. Red corresponds to leftward lateralization, whereas blue corresponds to rightward lateralization. **B.** Correlation of individual patterns of LI in each parcel with that of VTC, across participants, excluding parcels that overlap with the large VTC parcel. **C.** Scatter plot comparing the parcel-level mean LI with the across-participant correlation of LI between the parcel and VTC, along with a line of best fit and result of a spearman correlation across parcels. **D.** Results of **B.** masked to show only the 50 parcels with the largest mean laterality ($|LI|$).

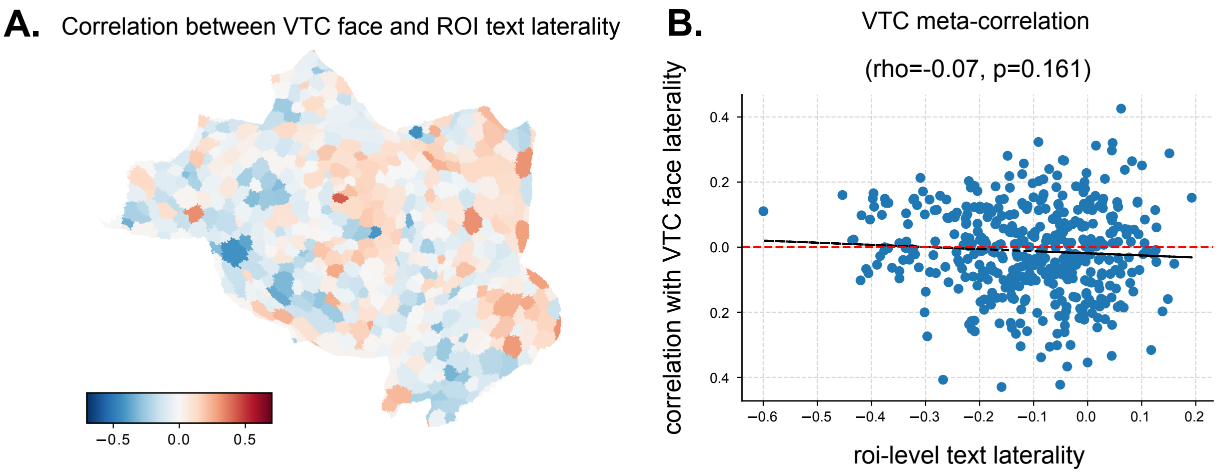

Figure S10: Data-driven assessment of whole-brain coupling of text selectivity with VTC face selectivity in the main experiment. **A.** Correlation of individual patterns of text LI in each parcel with face LI in VTC, across participants. **B.** Scatter plot comparing the parcel-level mean LI with the across-participant correlation of LI between the parcel and VTC, along with a line of best fit and result of a spearman correlation across parcels.

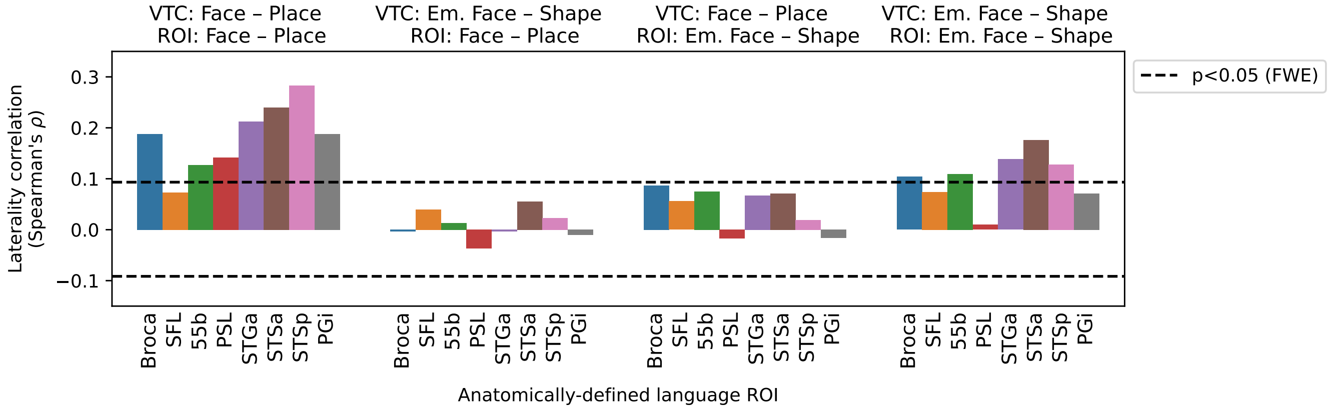

Figure S11: LI correlations across face tasks in VTC and Rajimehr language parcels. The same approach as in Figure 8 of the main text is used. Laterality coupling with VTC is significant in most parcels in both tasks, however coupling is significantly reduced when comparing across tasks. This suggests that face laterality coupling between VTC and language/social regions may be dependent on the degree of social or emotional processing inherent to the face processing task. One caveat to this result is that we were unable to construct independent subsets of data within each task, so long-range correlations may be partially driven by non-task-based co-fluctuations in the signal.

7 Reanalysis of in-house data including 4 excluded subjects

7.1 Distributions of laterality and overlap of selectivity

In the main analyses of our in-house experiment, two subjects were excluded due to showing rightward laterality of text selectivity (see Figure S6; subjects 4 and 50), and two subjects were excluded due to not completing the diffusion experiment (Figure S6; subjects 20 and 31). Additionally, we removed outliers from individual laterality comparisons, determined on the basis of being 3 standard deviations or more away from the mean. As these details were not reported in our methods until the proofing stage, here, we demonstrate the effects of these subject exclusion choices, which are very minimal with respect to the claims made in our paper.

First, in Figure S12, we compare distributions of laterality, consistency of laterality, and overlap of topographic selectivity, across both sets of subjects. The only notable difference is the presence of two abnormally right-lateralized subjects in the laterality distributions of Figure S12A and B. The overlap analyses are essentially unchanged.

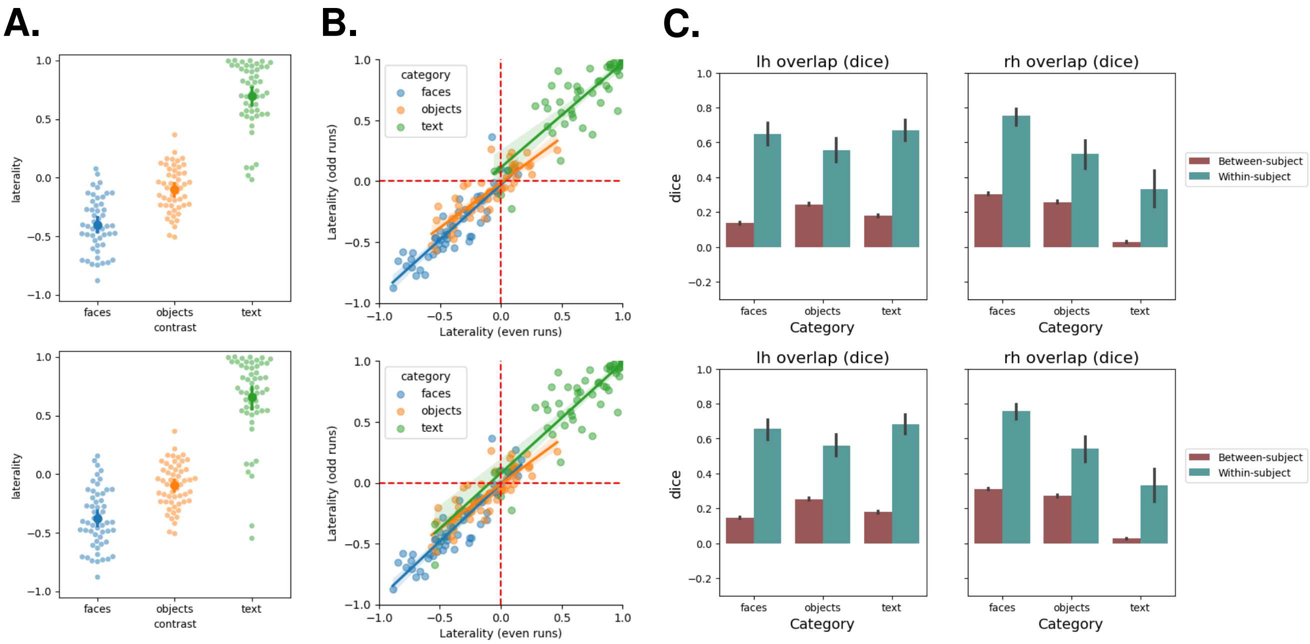

Figure S12: Reanalysis of Figure 4, comparing the set of subjects used in the main paper (top) with matched analyses using all 55 subjects (bottom). Each panel corresponds to the respective panel in Figure 4, and the top row simply replicates the analyses shown in the main paper.

7.2 Individual differences local to VTC

Next, we demonstrate the effects of our exclusion on individual difference analyses. Figure 5 is reproduced as Figure S13. In comparison to the matched analyses using all 55 subjects (Figure S14), only one significant difference emerges. The significance of two positive correlations between face and text laterality (held-out category baseline (2nd column) and fixation baseline with held-out category regressed out (fourth column)) are abolished when using all 55 subjects. The former correlation was not of major interest to us, since we argued it reflected category-based confounding. The latter correlation was of somewhat greater interest, and while its significance is abolished, the effect magnitude is similar ($r = 0.316$ vs. $r = 0.228$) and the effect is statistically trending ($p < .1$). The reduction in the correlation is directly a consequence of excluding the two right-lateralized subjects. If we include the two non-outlier subjects with missing diffusion data (Figure S15), the significance of this correlation is restored ($r = 0.313$, $p < 0.05$).

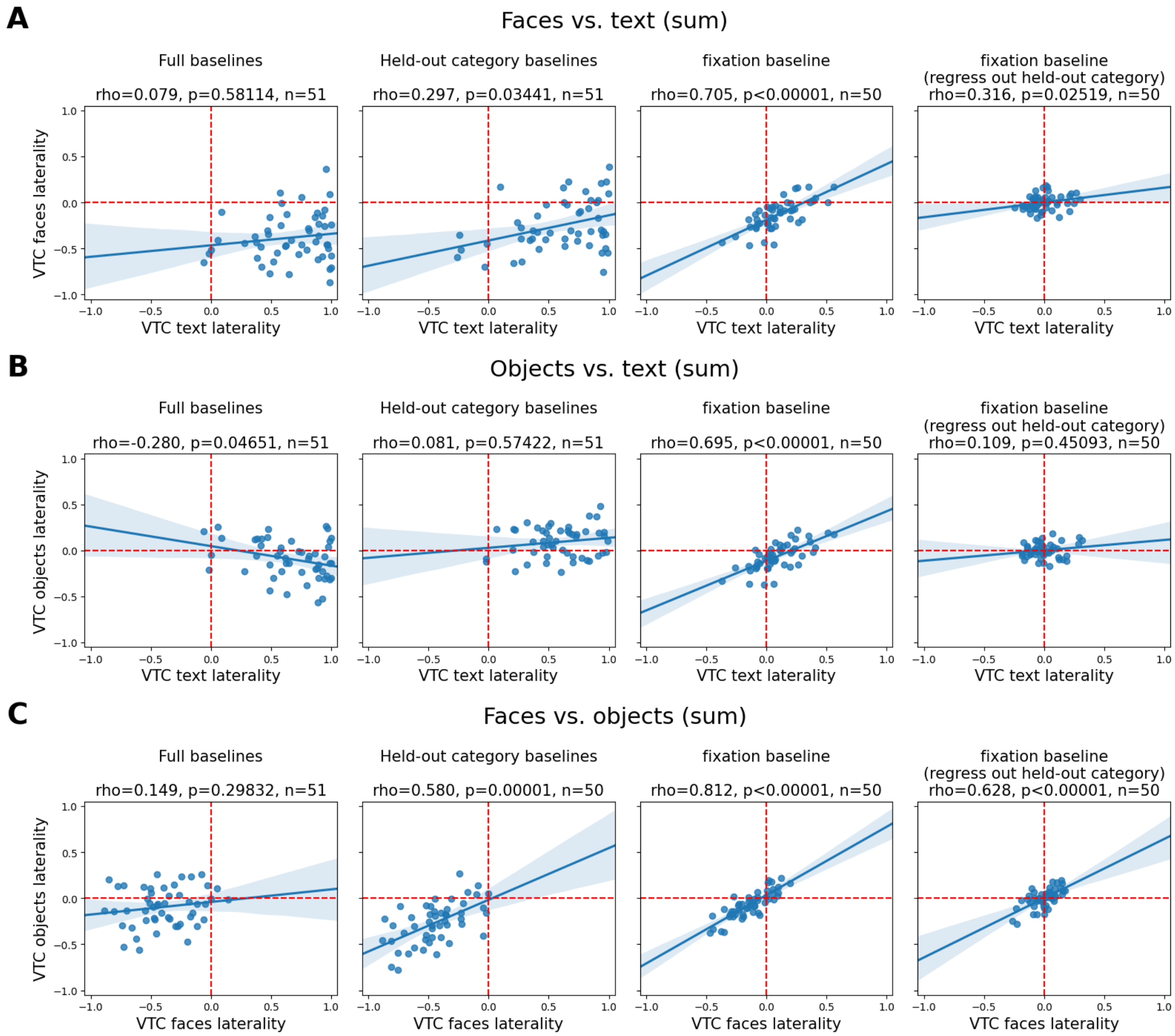

Figure S13: Replication of Figure 5 for convenience of comparison to Figure S14.

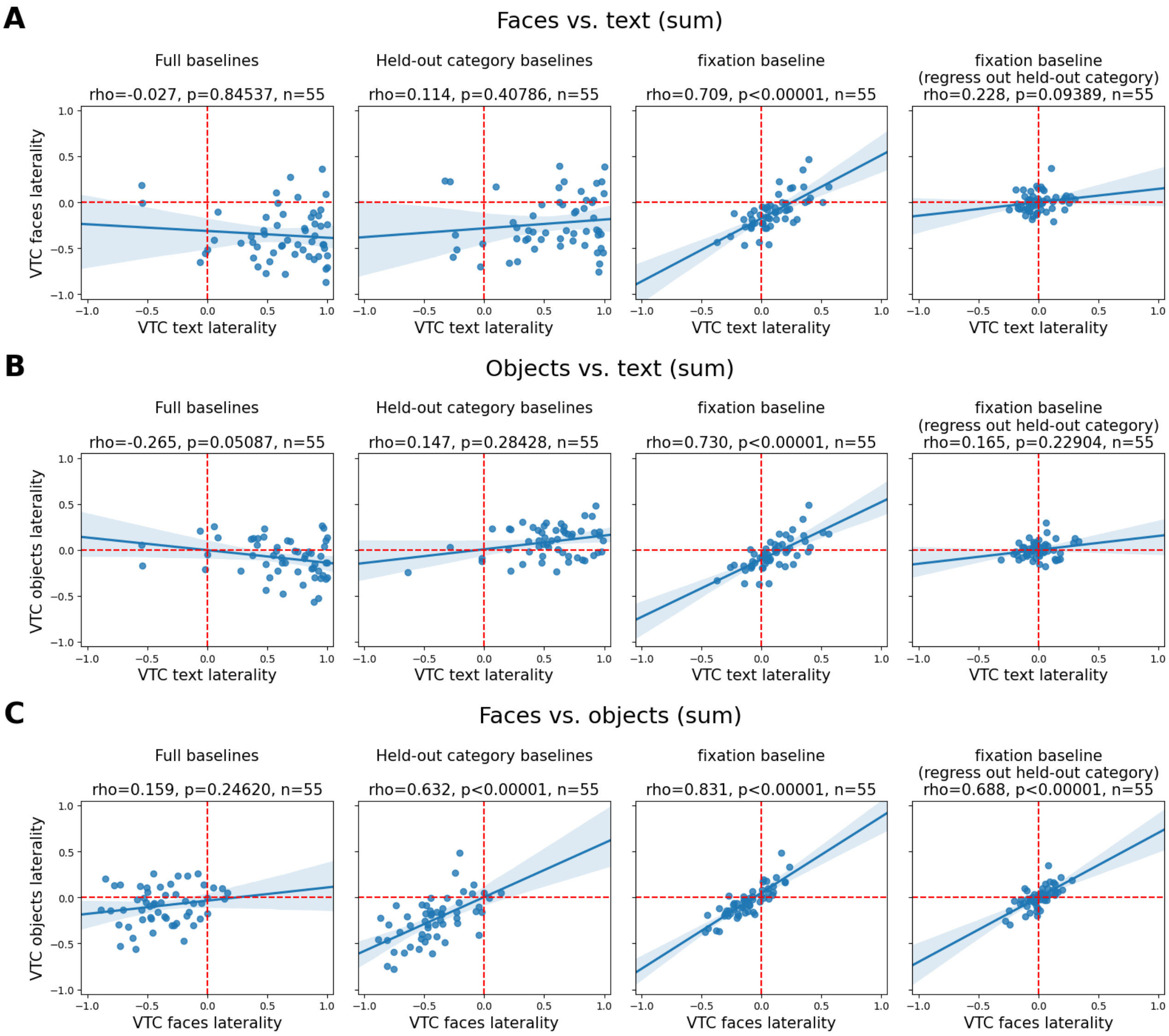

Figure S14: Reanalysis of Figure 5 using all 55 subjects.

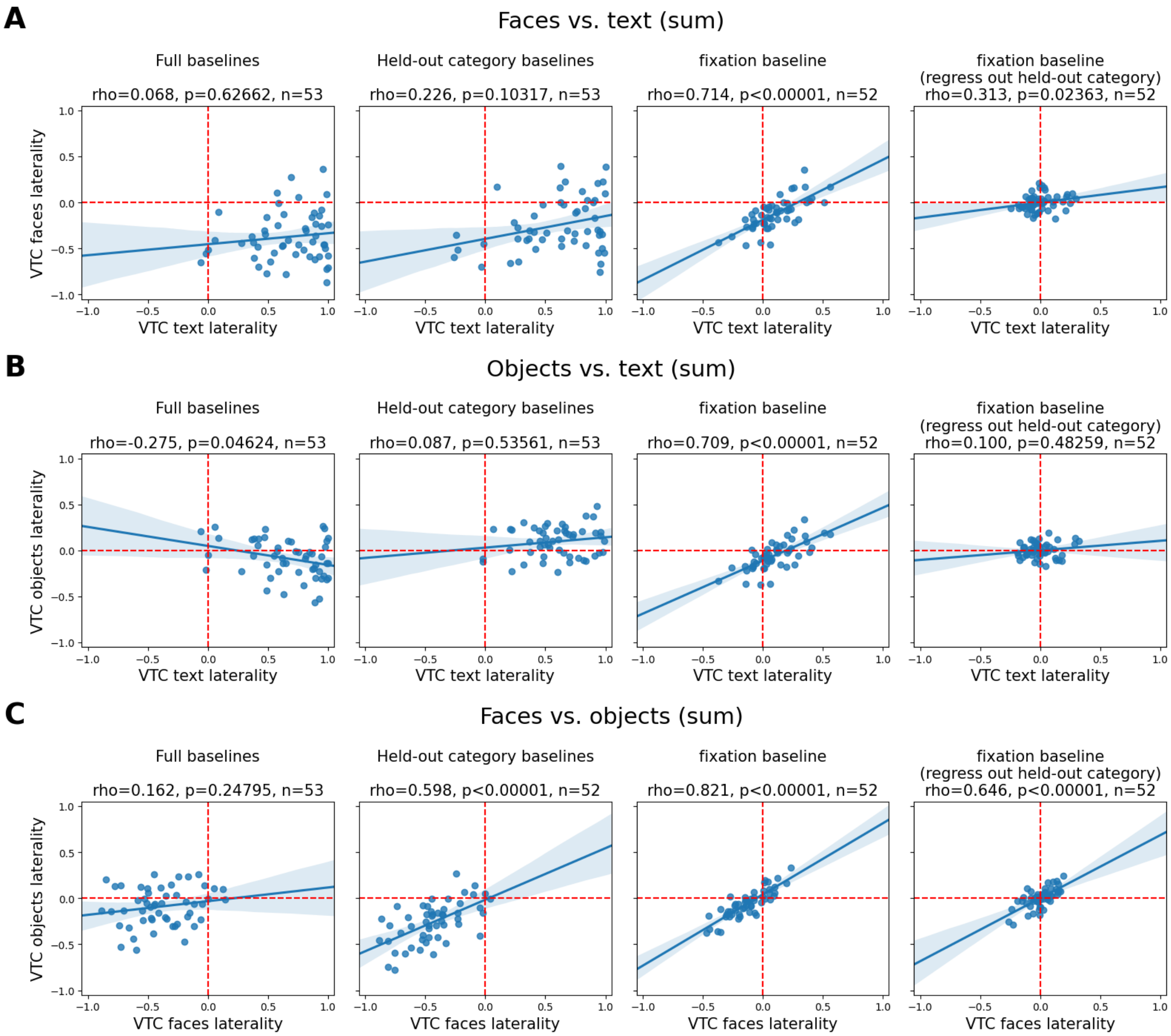

Figure S15: Replication of Figure 5 using 53 subjects without rightward text lateralization.

7.3 Individual differences in long-range coupling for VTC text processing

Last, we examine the long-range coupling analyses for text. We replicate Figure 7 in Figure S16, and perform the matched analyses with all 55 subjects in Figure S17. The ROI-level long-range coupling analyses for text (Figures S16, S17 panel A) are largely unchanged; indeed, if anything, the correlations are slightly increased with the presence of right-text-lateralized subjects, who tend to show bilateral or rightward text laterality in the other ROIs. Additionally, the parcel-level results (Figures S16, S17 panel B-E) are essentially unchanged.

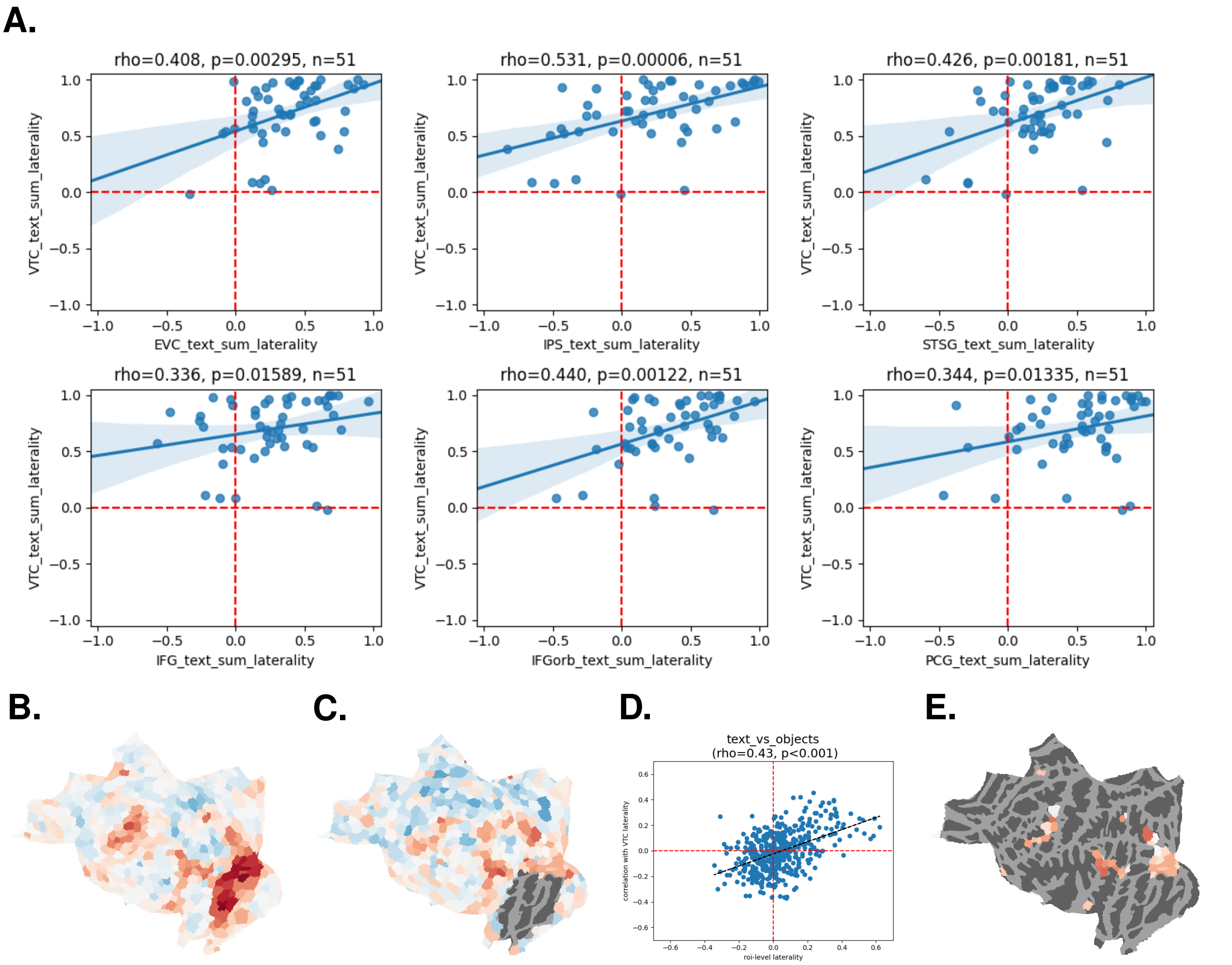

Figure S16: Replotting of Figure 7 from the paper. **A.** Laterality of VTC text selectivity correlated with laterality of text selectivity across this distributed network of text-activated regions. **B.** Mean of individual atlas parcels across participants. Red corresponds to leftward lateralization, whereas blue corresponds to rightward lateralization. **C.** Correlation of individual patterns of laterality in each parcel with that of VTC, across participants, excluding parcels that overlap with the large VTC parcel. **D.** Scatter plot comparing the parcel-level mean laterality with the across-participant correlation of laterality between the parcel and VTC, along with a line of best fit and result of a spearman correlation across parcels. **E.** Results of **B.** masked to show only the 50 parcels with the largest mean laterality. The vast majority of these lateralized parcels show an across-participant correlation in laterality with VTC.

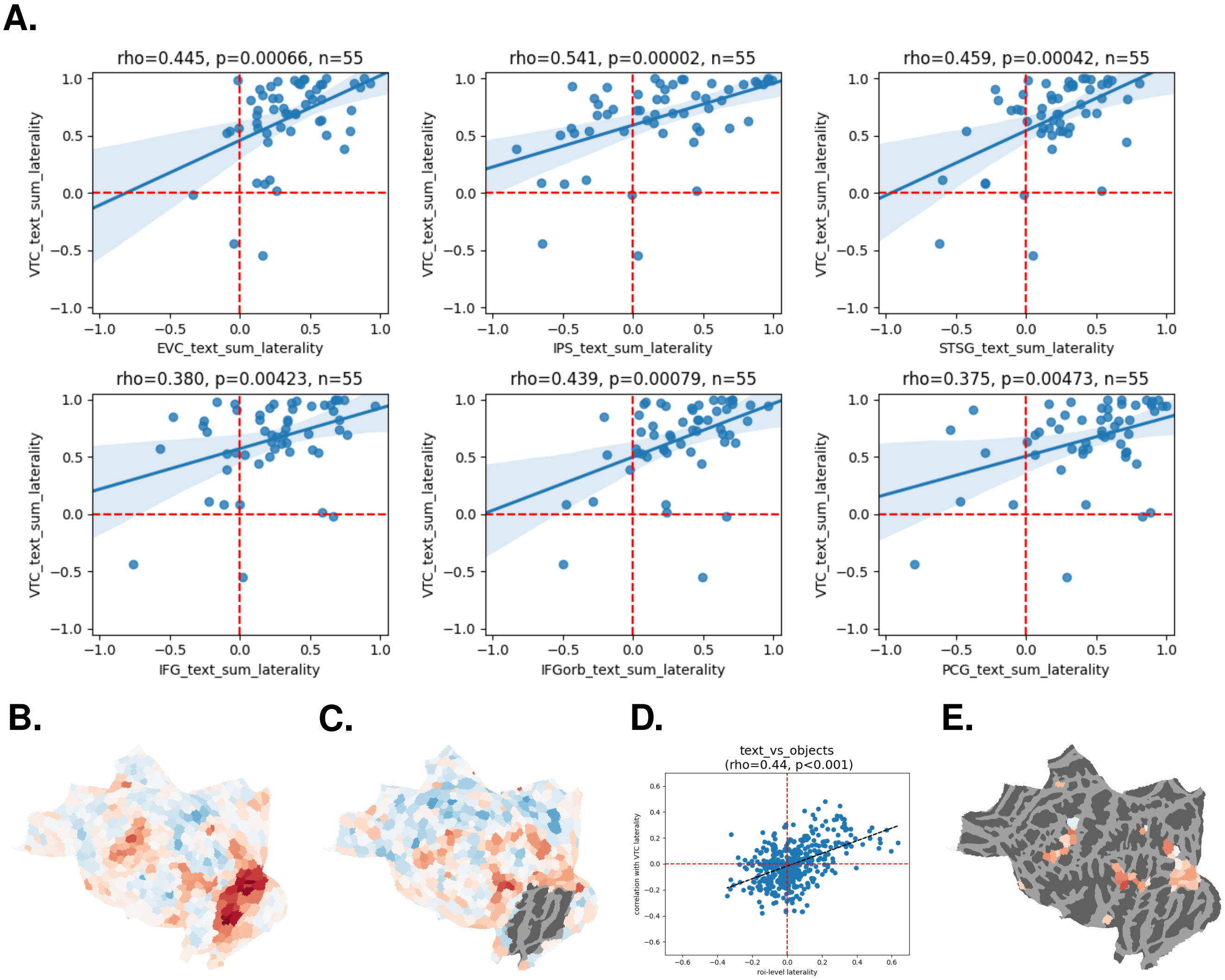

Figure S17: Reanalysis of Figure 7 using all 55 subjects (see caption of Figure S16).
